# Health management committee strengthening and community mobilisation through women’s groups to improve trained health worker attendance at birth in rural Nepal: a cluster randomised controlled trial

**DOI:** 10.1186/s12884-020-02960-6

**Published:** 2020-05-06

**Authors:** Joanna Morrison, Kirti Tumbahangphe, Aman Sen, Lu Gram, Bharat Budhathoki, Rishi Neupane, Rita Thapa, Kunta Dahal, Bidur Thapa, Dharma Manandhar, Anthony Costello, David Osrin

**Affiliations:** 1 Institute for Global Health, University College London, 30 Guilford Street, London, WC1N 1EH UK; 2 Mother and Infant Research Activities, PO Box 921, Thapathali, Kathmandu, Nepal

**Keywords:** Maternal, Newborn, Public accountability, South Asia, Participation, Health systems

## Abstract

**Background:**

Engaging citizens and communities to make services accountable is vital to achieving health development goals. Community participation in health management committees can increase public accountability of health services. We conducted a cluster randomised controlled trial to test the impact of strengthened health management committees (HMCs) and community mobilisation through women’s groups on institutional deliveries and deliveries by trained health workers in rural Nepal.

**Methods:**

The study was conducted in all Village Development Committee clusters in the hills district of Makwanpur (population of 420,500). In 21 intervention clusters, we conducted three-day workshops with HMCs to improve their capacity for planning and action and supported female community health volunteers to run women’s groups. These groups met once a month and mobilised communities to address barriers to institutional delivery through participatory learning and action cycles. We compared this intervention with 22 control clusters. Prospective surveillance from October 2010 to the end of September 2012 captured complete data on 13,721 deliveries in intervention and control areas. Analysis was by intention to treat.

**Results:**

The women’s group intervention was implemented as intended, but we were unable to support HMCs as planned because many did not meet regularly. The activities of community based organisations were systematically targeted at control clusters, which meant that there were no true ‘control’ clusters. 39% (5403) of deliveries were in health institutions and trained health workers attended most of them. There were no differences between trial arms in institutional delivery uptake (1.45, 0.76–2.78) or attendance by trained health workers (OR 1.43, 95% CI 0.74–2.74).

**Conclusions:**

The absence of a true counterfactual and inadequate coverage of the HMC strengthening intervention impedes our ability to draw conclusions. Further research is needed to test the effectiveness of strengthening public accountability mechanisms on increased utilisation of services at delivery.

**Trial registration:**

Current Controlled Trials ISRCTN99834806.

Date of registration:28/09/10.

## Background

### Public accountability of healthcare institutions

There is increasing interest in public accountability mechanisms to promote people-centred health care, improving quality and equity [1]. Communities can increase accountability through exercising ‘exit’ strategies, through which individuals can access alternative suppliers, or ‘voice’ strategies, which relate to their capacity to exert pressure on the health service to perform [2]. Structures that can enable ‘voice’ strategies include citizens’ juries [3], health management committees (HMCs), regular social audits [4], and other active linkages between health facilities and community groups.

Despite international interest in public accountability, there is little evidence about the effectiveness of accountability mechanisms on health outcomes. A review found that few studies presented good quality quantitative data using observable measures of impact [5].

### Maternal and newborn health in Nepal

Nepal’s maternal and neonatal health indicators are improving, yet many women still deliver at home, particularly in rural areas, without access to a trained health worker at delivery [6]. Significant policy responses to improve maternal and newborn health include providing incentives to women and service providers for institutional delivery through the *Aama Suraksha Karyakram* (safe motherhood programme) [7], upgrading primary healthcare facilities to become birthing centres, implementing a national skilled birth attendant strategy with a short-term plan of training for nurses [8], and making finances available at district level for local recruitment of nurses at newly established birthing centres [9].

### Engaging citizens and communities in Nepal

The Nepal health sector strategy recognises the need to engage citizens and communities to make services and service providers accountable [10]. Recent implementation of federalism and decentralisation of health system governance to newly elected officials in municipalities at the end of 2017 presents an opportunity to improve public accountability. Guidelines for existing governance structures such as the Health Facility Management Committees have not yet been revised. The handover of health facilities to local management committees began in 2004 and operational guidelines were finalised in 2010 [11]. There is one health facility and HMC per 9000 population. HMCs are usually chaired by the local political representative, the Village Development Committee (VDC) Secretary. Other members are the health facility in-charge and community representatives, who should include at least one member of a marginalised ethnic or caste group. A Female Community Health Volunteer (FCHV) is also often a member. HMCs should meet at least once a month and seek to improve quality of care through monitoring, evaluation and planning. Training and orientation is being implemented at a national level, but few mechanisms are in place to provide sustained support to HMCs in the long term. Appreciative Inquiry (AI) approaches are popular in Nepal, and have been used by the Government of Nepal to support innovation and team development in health management committees [12]. AI is an approach to nurture organizational change which is based on positive psychology and has been used in the business, health and community-development sectors [13]. It starts from the premise that change occurs through discovering and valuing the strengths and ideas of people in an organisation. In contrast with problem solving approaches, it is a technique that examines what is working well within an organisation and seeks to amplify and replicate these attributes [14]. AI approaches follow a four ‘D’ cycle in which participants ‘discover’ and articulate their strengths and core values through recalling a rewarding experience or a time when the organisation was most effective. Through this process of discovery, participants recognise what gives them purpose and can imagine a preferred vision or ‘dream’ of what the organisation could be. Participants visualize what their practice might be in this ‘dream’ phase, and common themes for preferred future practice are identified. Next, participants discuss the steps necessary to realise their dream in the ‘design’ phase, which can involve a process of prioritization and the formation of action teams to focus on tasks [15]. The next phase, ‘destiny’, is a chance to review progress, to celebrate achievements and learning, and to validate actions. This phase is reflective and can be a basis from which to work through the AI cycle again, considering how actions fit with the vision of the future, celebrating past successes and applying skills learned to new issues. Although there is no evidence base for the effectiveness of this approach, its ubiquity and potential to build on the positive was appealing to our research team.

At the community level, public accountability can also be improved through participation of community groups in service planning and delivery. In Nepal, FCHVs are mandated to increase community participation to improve local health by convening women’s groups in every VDC [16]. They are tasked with running one women’s group per month to raise awareness about health issues. FCHVs are an important link between the community and the health facility and some feel that they have made a significant contribution to Nepal’s improved maternal and child health outcomes [17–19]. The successful operation of women’s groups is variable and depends on the capacity of FCHVs and the degree of support they receive.

The Institute for Global Health, University College London, and Mother and Infant Research Activities (MIRA), Nepal, were part of a research consortium testing the effectiveness of participatory women’s groups on newborn mortality in low-income countries. A meta-analysis of consortium cluster randomised controlled trials has shown that participatory interventions with women’s groups can reduce newborn mortality by up to 49% if at least 33% of pregnant women participate in the groups [20]. The meta-analysis also showed improvements in maternal health in women’s group clusters. Our previous study in Makwanpur District employed local female facilitators to convene groups and guide them through a participatory action cycle. Field supervisors provided ongoing support and mentored facilitators. A cluster randomised controlled trial showed a 30% reduction in newborn mortality in clusters with women’s groups compared with control clusters, and small but significant increases in institutional deliveries [21]. Nonetheless, most women still delivered at home.

We hypothesized that a synergistic approach that worked with existing national public accountability structures – FCHV-run women’s groups and HMCs - would increase uptake of institutional deliveries and deliveries with a trained health worker. We present findings from a cluster randomised controlled trial testing the effect of health management committee strengthening and community mobilisation through women’s groups on institutional deliveries and deliveries by trained health workers [22].

## Methods

### Setting

The study was implemented in the hill district of Makwanpur in central Nepal. It has a population of ~ 420,500 [23] and its Human Development Index score of 0.497 is slightly above the national average of 0.458 [24]. 83% of the population are engaged in agriculture and almost half are of Tibeto-Burman descent [23]. Twenty out of 43 VDCs are accessible by road year round, and 13 are inaccessible during the monsoon [25]. Private healthcare use is low, and government health services are provided through four Primary Health Care centres, nine Health Posts, and 30 Sub Health Posts.

### Trial design

We used a cluster randomised design because the intervention targeted families, communities, HMCs and government health workers. It was implemented in Makwanpur District, which has 43 geopolitical VDCs. We compared 21 intervention VDC clusters with 22 control clusters.

### Participants

The sampling frame included women aged 12 to 49 years who delivered infants between 1st October 2010 and 30th September 2012.

### Allocation

**Fig. 1:**
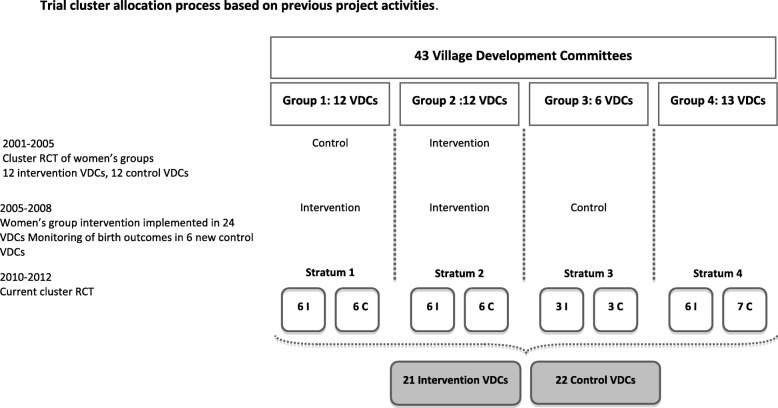
Trial Cluster Allocation

We allocated clusters to intervention or control at a public meeting. Clusters were stratified into four groups according to their previous exposure to women’s group activities (Fig. 1). From 2001 to 2005 12 intervention VDCs received a women’s group intervention and 12 VDCs served as controls as part of a cluster randomised controlled trial [21]. From 2005 to 2008, the women’s group intervention was implemented in all 24 VDCs - 12 intervention and 12 previous control VDCs - and birth outcomes were monitored in six additional VDCs. Within each stratification, equal numbers of clusters were randomly allocated to receive the intervention using a lottery method.

### Outcomes

#### Primary

We measured the effect of the intervention on two primary indicators: deliveries conducted by a doctor, nurse or auxiliary nurse midwife (trained health workers), and institutional deliveries that occurred at a Sub Health Post, Health Post, Primary Health Centre, hospital or private health institution.

#### Secondary

Secondary outcomes included uptake of antenatal care (four or more consultations) and postnatal care. Postnatal care was defined as any consultation with a health worker within 6 weeks of delivery that was not primarily for infant immunisation. We also report neonatal deaths per 1000 live births, and stillbirths per 1000 births, as these were measured during previous interventions and we were interested in observing incremental changes.

#### The intervention

##### The HMC intervention

**Fig. 2:**
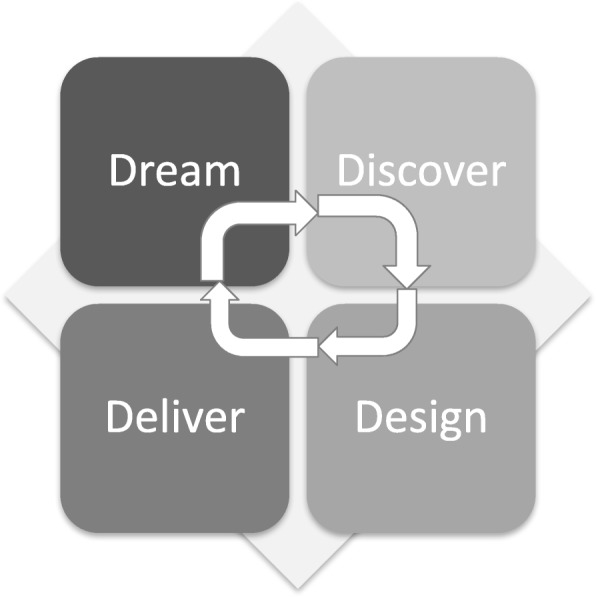
4 ‘D’ Cycle of Appreciative Inquiry

The HMC strengthening intervention intended to activate HMCs using the principles of Appreciative Inquiry [15]. We ran four-day workshops at each health facility in intervention areas from June 2010 to September 2010. HMC members (177), community representatives (202), health workers and auxiliary staff (54) worked through the first three stages of a ‘four D’ cycle (Discovery, Dream, Design, Deliver) (Fig. 2). We supported HMCs through the last stage of the cycle over the next 2 years.

We had intended intervention supervisors to meet with HMC members at each health facility every two or 3 months at their regular meeting, to encourage them and monitor their progress. Unfortunately, this was not possible because of difficulties in coordinating meetings between researchers and busy HMC members. Therefore, three HMCs each received four follow-up meetings, 14 HMCs each received three follow-up meetings, and four HMCs each received one follow-up meeting over the two-year intervention period. One of these meetings was a cluster review meeting conducted 1 year after the workshop. All initial workshop participants were invited to discuss progress towards dreams. We also facilitated a one-day District review workshop at which member secretaries and chairpersons from each HMC met to share progress and learn from others.

##### Women’s group intervention

We trained 195 FCHVs in facilitation skills, the participatory learning and action cycle process, and how to run meetings. They were given a manual containing discussion points, games and stories, and had additional orientation about content at monthly supervision meetings. Seven supervisors supported 20–30 FCHVs each. One hundred ninety-five FCHVs ran a total of 203 women’s groups per month, with eight conducting two meetings per month. FCHVs led women’s group discussions about barriers to institutional delivery and ways to address them. The group then organised community and cluster meetings to galvanise support for strategy implementation, to which HMCs, health workers, community leaders, women and men were invited. After strategies to address barriers were implemented, the group reflected on their progress and planned and implemented further strategies or changed existing ones. Women’s groups ran from March 2010 to September 2012.

### Process evaluation methods

We used a realist evaluation framework to consider the interaction of the intervention in context, triggering a change mechanism that affects outcomes [26]. Broadly, we sought to increase links between communities and health facilities. More specifically, we hypothesised that perceptions about the quality of care provided at health institutions would change as a result of the intervention. We collected data about intervention and control area context, implementation of the intervention and quality of care as a potential mechanism of change. We described context through baseline and endline surveys of the status and activities of health facilities, HMCs, active women’s groups, community based organisations (CBOs), and FCHVs. We also conducted two qualitative studies, one exploring reasons for home delivery [27], and one exploring perceptions about quality of care in intervention and control areas. To describe the women’s group intervention implementation, we analysed monitoring forms used by intervention supervisors when they observed women’s group meetings, and at monthly supervision meetings with FCHVs. Supervisors visited an average of 15 women’s groups per month. These were usually groups that needed more support. We collected quantitative and qualitative data about group attendance, the abilities of FCHVs, how interested the group members appeared, the extent to which the manual agenda was followed, factors hindering the process of the meeting, and specific information (for example, which problems were prioritised). We collected information about the number of meetings conducted, reasons for postponement, difficulties and suggestions for improvement of the manual. FCHVs also submitted a pictorial reporting form about the composition of the group at monthly meetings. Supervisors collected data about HMCs and their progress towards dreams. We documented dreams and plans at the initial workshop, and we collected data on the reasons for progress or lack of progress at review meetings. The endline survey of intervention and control areas described the status of HMCs and their activities over the past 2 years. Process evaluation officers conducted focus group discussions with supervisors at midline and endline, and conducted narrative observations of two women’s groups throughout the trial.

### Sample size

We estimated that each of 43 clusters would yield 100 annual births, and that 20% of births were attended by trained health workers at baseline. We estimated sample size using the equations of Hayes and Bennett [28], assuming two treatment groups and unmatched clusters of approximately equal size. We set a value of k - the between-cluster coefficient of variation - equal in intervention and control groups, and added 2 clusters to the estimated cluster number required to account for loss of degrees of freedom as a result of stratification. Estimates were based on a two-tailed 5% significance level and a range of k from 0.25 to 0.35. For 2 years of intervention (200 births per cluster), at k = 0.35, the sample would detect an increase in trained attendance from 20 to 28% at 80% power (from 20 to 26% at k = 0.25).

### Surveillance and data management

We ran a surveillance system that incentivised local women to identify births, newborn deaths, infant deaths, under five deaths, and deaths of women between 12 and 49 years. These women reported events at a monthly meeting with cluster interviewers, who verified them and administered a paper questionnaire. Supervisors observed 10% of interviews and questionnaires were checked on site and in the field office to ensure data quality. Data were entered through a Visual Basic interface into a relational database management system in Microsoft SQL server 2007. 10% of questionnaires were double-entered and an error rate of < 3% was considered acceptable.

### Analysis

We compared control and intervention groups in terms of frequencies and proportions of demographic, socioeconomic and outcome indicators at baseline. Primary and secondary outcomes referred to pregnancies and deliveries. We tabulated frequencies and proportions of outcome indicators by allocation. Given the lack of baseline imbalance, differences in outcomes between control and intervention arms were evaluated through univariable logistic regression models, including a random effect for cluster.

### Ethical concerns

#### Health service strengthening

Irrespective of allocation, 154 health workers in all clusters and the district headquarters received three-day refresher training on emergency obstetric care. Following training and health facility audit, we supplied 22 locally made neonatal resuscitaires to birthing centres with an electricity source. We also supplied poster protocols for normal deliveries, shock management, Apgar scoring, neonatal resuscitation and medication to birthing centres. A limited number of magnesium sulphate protocols and commonly used drugs were also distributed.

The Nepal Health Research Council (Ref: 889) and University College London Research Ethics Committee (2257/001) approved the study. Cluster representatives gave written informed consent for study implementation. Informed verbal consent was taken from individual women due to high levels of illiteracy, and consent was also taken from a guardian where the participant was under the age of 16 years old. These consent procedures were approved by both ethics committees. A Data Monitoring Committee meeting in November 2011 recommended that the trial be completed as intended. A final Data Monitoring Committee was convened in January 2012. Our study adheres to CONSORT guidelines.

## Results

### Process evaluation: HMC intervention

We conducted 21 workshops with the help of District Public Health Office personnel who had received training in the AI approach. The most common dreams documented were to ensure that the HMC remained active, to increase community health awareness, to improve infrastructure, and to procure equipment (Table 1). It was often unclear how progress towards far-reaching dreams would be measured (raising awareness, for example). An average of eight dreams were proposed for each HMC.

**Table 1:** Health Management Committee prioritised dreams

Dream	HMC dreams	HMC dreams achieved
Keep the HMC active	18	14
Increase health awareness in the community	18	12
Improve physical infrastructure	16	7
Procure equipment for maternal and newborn care	12	4
Upgrade the health facility (for example make an SHP an HP)	11	2
Provide 24-h/effective delivery services	10	9
Recruit an Auxiliary Nurse Midwife	10	8
Maintain a user friendly environment in the health facility	8	3
Start providing delivery services	8	5
Fill vacant posts	6	1
Buy an ambulance	8	1
Improve cleanliness and the physical environment of the health facility	6	2
Monitor and reward personnel and co-ordinate with other organisations	6	2
Ensure the mobilisation of the FCHV	5	3
Increase quality of health services	5	1
Monitor and maintain regular attendance of staff and maintain opening hours	4	2
Improve maternal and newborn health	4	2
Register land in the name of the health institution	3	0
Provide more services	3	2
Conduct a family planning campaign	2	2
Team building training for HMCs and health personnel	2	1
Increase financial transparency	1	1
Put up a notice board to track numbers of institutional deliveries	1	1

**Fig. 3:**
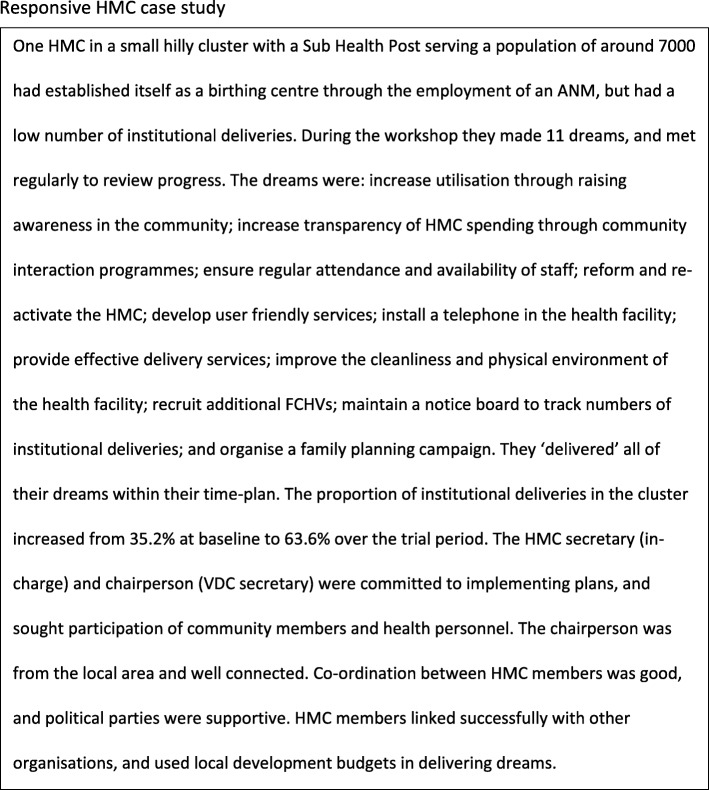
Responsive HMC case study

**Fig. 4:**
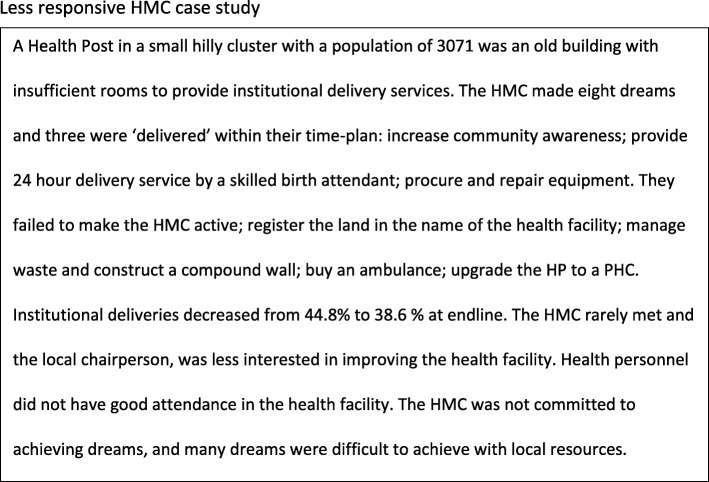
Less responsive HMC case study

Some HMCs became very active, and the workshops catalysed action (Fig. 3). Dreams were more difficult to achieve when the HMC was not active, did not meet regularly, had a disinterested chairperson and intermittent attendance of health personnel HMCs also found it challenging when dreams were difficult to achieve with local resources (Fig. 4). Most HMCs had meetings infrequently and irregularly, usually because it was difficult to arrange a time when all members were available. Instead of having a regular meeting they met ‘as and when necessary’. These meetings were arranged without informing intervention supervisors because they were ad hoc; or this may have been a strategy to prevent supervisors from participating in internal planning and budgeting. Supervisors also supported FCHV women’s groups and found it difficult to attend HMC meetings at short notice. They met informally with individual HMC members and discussed progress, but this was not done systematically. As it was usually infeasible to meet at HMC meetings, supervisors requested the HMC secretary to call meetings to discuss progress toward dreams. These requests were again difficult to fulfil because the HMC chairperson was often too busy to attend.

### Process evaluation: Women’s group intervention

Six thousand forty-one group meetings were held over 32 months. FCHVs maintained an average of 181 meetings per month over the trial period. On average, each group had 16 women attending, one pregnant woman and two men. In intervention areas, the proportion of pregnant women attending FCHV group meetings was 14.3% (896), compared with 4.7% (353) in control areas. The barriers to institutional delivery identified by women’s groups were similar to those identified in our qualitative study, although women’s groups reported ‘use of traditional healer’ which was given less emphasis in the qualitative study (Table 2).

**Table 2:** Barriers identified by groups

Barriers to institutional delivery	Groups (***n*** = 203)
Lack of money	104
Embarrassment and fear	98
Lack of knowledge	89
Belief in traditional healers	81
Lack of family support	75
Lack of 24-h service	71
Lack of transportation	62
Geographic difficulties	52
Absence of health workers	20

Group strategies usually targeted more than one barrier (Table 3). To address family barriers to institutional delivery, many groups started a referral or invitation card system for antenatal care and institutional delivery. Group members visited pregnant women in their homes and gave them formal invitation cards from the health facility.

**Table 3:** Strategies to address barriers to institutional delivery

Strategy to address barriers	No. groups that suggested this strategy	No. groups that implemented this strategy	No. of groups that evaluated this strategy
*First action-learning cycle*^a^			
Awareness programme	71	172	172
Counselling of pregnant women & family	143	123	123
Mutual fund	93	163	163
Information about AMA programme & danger signs	75	60	60
Stretcher scheme	73	113	113
Interaction with traditional healers	70	19	19
Advocacy at the local health institution	9	10	10
*Second cycle*^a^			
Referral card	167	167	n/a^b^
Invitation card to pregnant women & household head	159	159	n/a^b^
Mobile meeting	95	95	n/a^b^

^a^refers to the participatory action-learning cycles that women’s groups engage in

^b^were not completed within the timeframe of the trial

Groups held 17 out of a potential 21 cluster meetings, which involved the HMC, women’s groups from the cluster, and other community members. One hundred ninety-nine community meetings were held out of a potential 203. Of 465 meetings observed by supervisors, 17% (78) felt that the FCHV was very active and motivated. Only 13% (60) of observed meetings were fully participatory, and 27% (123) of FCHVs needed substantial help conducting the meeting. The capacity of the FCHVs affected intervention implementation. Older, illiterate FCHVs were reluctant to follow the meeting agenda and did not initiate participatory activities, games, or role-play. They found the intervention more difficult to implement, and younger, better-educated and enthusiastic FCHVs were more successful at running groups and mobilising communities (focus group discussion endline). Supervisors felt that, overall, FCHV skills improved over time. The proportion who found meetings difficult to conduct decreased from 29% (57) in the problem identification stage to 11% (22) in the second implementation cycle.

Supervisors often found it difficult to support FCHVs adequately. Meetings were arranged according to community convenience, and most were conducted in the first week of the month. These factors, combined with the hilly topography of the district, remoteness of communities, and the high number of clusters that each supervisor had to support, resulted in limited supervision of FCHVs. Despite the manual containing many pictorial aids, supervisors felt that more pictorial instruction would be beneficial. The first cycle strategy implementation was difficult for FCHVs to facilitate and for supervisors to oversee. The manual was less detailed in this cycle, as meeting agendas were intended to follow-up on different commitments and plans made at community meetings. Monthly orientation meetings became difficult to manage because of the differing agendas of each group. This led to similar strategies being implemented by groups. Some groups were delayed in implementing strategies and did not have time to evaluate them. These groups evaluated their own performance generally, instead of the performance of strategies. Some groups found it difficult to implement strategies because the FCHV had been changed, was living in another community, or was not interested in conducting the meeting.

### Process evaluation: context

There were more community-based organisations (CBOs) working in control areas than intervention areas at baseline and endline. This was partly a result of the District Development Committee recommendation that other CBOs working in reproductive health focus on HMC strengthening and women’s group activities in control clusters, to avoid duplication of efforts and achieve district-wide coverage of interventions. At baseline, FCHVs in control areas were slightly younger, and by the end of the trial only 9% of FCHVs in control areas were illiterate compared to 22% in intervention areas. There were also more private clinics and medicine shops in control areas compared to intervention areas at baseline and endline (Table 4).

**Table 4:** Process evaluation: Context

	Baseline		Endline	
	Intervention n (%)	Control n (%)	Intervention n (%)	Control n (%)
**Community based organisation activities**				
Pregnant women’s group (Centre for Community Development Nepal (CCDN))	16	25	78	81
Women’s group (District government Women and Children Office)	11	34	60	124
Health Management Committee strengthening (CCDN)	0	0	3	6
**FCHV status**				
18–35	66 (46)	110 (54)	100 (52)	103 (50)
36–55	85 (49)	102 (36)	88 (46)	86 (42)
55 and above	9 (5)	21 (10)	4 (2)	17 (8)
Illiterate	48 (28)	59 (29)	42 (22)	18 (9)
Functioning FCHV women’s group	133 (65)	160 (78)	191 (94)	159 (77)
**HMC status**				
Received an orientation of their roles and responsibilities	8 (38)	7 (31)	20 (95)	16 (72)
The VDC secretary was chairperson	11 (52)	10 (46)	9 (43)	14 (64)
Conducted regular meetings	7 (33)	8 (36)	13 (62)	11 (50)
HMC had conducted a community interaction programme	1 (5.6)	0 (0)	12 (57)	6 (27)
Initiated at least four activities in the past year	10 (48)	8 (36)	17 (80)	16 (72)
HMC meetings not run because of absent members	–	–	4 (19)	7 (32)
Absence of all or some members from HMC meeting due to personal workload	8 (38)	8 (36)	13 (62)	13 (59)
**Health workers involved in delivery services**				
Doctor	2	2	2	2
Staff nurse	1	2	1	2
ANM	17	20	33	33
MCHW	16	15	14	12
**Health workers with SBA training**				
Staff nurse	–	–	–	1
ANM	1	1	8	7
MCHW	–	–	2	1
**Vacant Posts**				
Staff nurse	–	1	1	–
ANM	1	1	2	–
MCHW	–	2	–	1
**Nurses on short term contracts**				
ANM (DHO recruited)	4	3	13	14
ANM (HMC recruited)	7	10	9	6
**Health facilities providing 24 h delivery care**	6	7	17	15
**Inj Magnesium Sulphate 2 cc/1 g (for eclampsia treatment)**	8	6	13	13
**Placenta pit**	0	0	7	9
**Private clinic or medical shop**	38	63	55	68

### Baseline data

Table 5 shows a baseline comparison of trial arms, using data collected from 19th November 2009 to 30th September 2010 from women identified through the newborn surveillance system. Control clusters had a median 121 households each (interquartile range 89–217) and intervention clusters 97 (71–134). Family size was a median 7 (7–8) in both groups.

**Table 5:** Baseline comparison of allocation groups, November 2009 – September 2010

	Control n (%)	Intervention n (%)	All n (%)
**Households**	**3468 (100)**	**2818 (100)**	**6286 (100)**
Agricultural livelihood	3093 (89.2)	2612 (92.7)	5705 (90.8)
Own land	3261 (94.0)	2706 (96.0)	5967 (94.9)
Own home	3349 (96.6)	2716 (96.4)	6065 (96.5)
Mud and stone walls	1939 (55.9)	1641 (58.2)	3580 (57.0)
Zinc roof	1512 (43.6)	1117 (39.6)	2629 (41.8)
Mud floor	3021 (87.1)	2625 (93.2)	5646 (89.8)
Electric light	1707 (49.2)	1600 (56.8)	3307 (52.6)
Woodburning stove	3359 (96.9)	2739 (97.2)	6098 (97.0)
Public wellwater	2275 (65.6)	1881 (66.7)	4156 (66.1)
Bush toilet	2230 (64.3)	1890 (67.1)	4120 (65.5)
*Ethnic identity*			
Tamang	2283 (65.8)	1609 (57.1)	3892 (61.9)
Brahmin-Chhetri	383 (11.0)	382 (13.6)	765 (12.2)
Praja	306 (8.8)	274 (9.7)	580 (9.2)
Magar	138 (4.0)	93 (3.3)	231 (3.7)
Other	358 (10.3)	460 (16.3)	818 (13.0)
*Socioeconomic position*			
Asset quintile 1	975 (28.1)	754 (26.8)	1729 (27.5)
Asset quintile 2	628 (18.1)	602 (21.4)	1230 (19.6)
Asset quintile 3	455 (13.1)	384 (13.6)	839 (13.3)
Asset quintile 4	661 (19.1)	570 (20.2)	1231 (19.6)
Asset quintile 5	749 (21.6)	508 (18.0)	1257 (20.0)
Women who delivered*	**3521 (100)**	**2853 (100)**	**6374 (100)**
*Age*			
< = 19 y	585 (16.6)	499 (17.5)	1084 (17.0)
20–29 y	2273 (64.6)	1813 (63.5)	4086 (64.1)
> =30 y	663 (18.8)	541 (19.0)	1204 (18.9)
Primiparous	1118 (31.7)	872 (30.6)	1990 (31.2)
*Schooling*			
None	1698 (48.2)	1426 (50.0)	3124 (49.0)
Primary	1008 (28.6)	800 (28.0)	1808 (28.4)
Secondary or higher	815 (23.2)	627 (22.0)	1442 (22.6)
*Reading*			
Cannot read	1457 (41.4)	1217 (42.6)	2674 (42.0)
Reads with difficulty	640 (18.2)	490 (17.2)	1130 (17.7)
Reads with ease	1424 (40.4)	1146 (40.2)	2570 (40.3)
Primary outcomes**	**3524 (100)**	**2853 (100)**	**6737 (100)**
Institutional delivery	1069 (30.3)	848 (29.7)	1917 (30.1)
Home delivery	2455 (69.7)	2005 (70.3)	4460 (69.9)
Institutional delivery conducted by doctor, nurse or auxiliary nurse midwife	1031 (29.3)	819 (28.7)	1850 (27.5)
Home delivery conducted by doctor, nurse or auxiliary nurse midwife	17 (0.005)	11 (0.004)	28 (0.004)
Any delivery conducted by doctor, nurse or auxiliary nurse midwife	1048 (29.7)	830 (29.1)	1878 (29.4)
Secondary outcomes**	**3524 (100)**	**2853 (100)**	**6737 (100)**
> =4 antenatal care visits	2374 (67.4)	1955 (68.5)	4329 (67.9)
Postnatal care visit	1595 (45.3)	1497 (52.4)	3092 (48.5)
**Attended FCHV women’s group**	**241 (6.8)**	**371 (13.0)**	**612 (9.6)**

* Outcomes reported per woman who had delivered. 3 women delivered twice between Nov 2009 and Sep 2010, in these cases, we reported values for all outcomes from the first delivery. ** Outcomes reported per delivery

Socio-demographic characteristics were similar in both arms, although there were more households in control clusters. Socioeconomic indicators were also similar in each arm, apart from intervention clusters having slightly more households with electricity (56%) than control (49%). The arms had comparable numbers of primiparous women, women with no schooling and women who had previously delivered in an institution. At baseline, 13% of women in intervention clusters and 7% of women in control clusters had attended FCHV women’s groups.

### Impact evaluation: participant flow

**Fig. 5:**
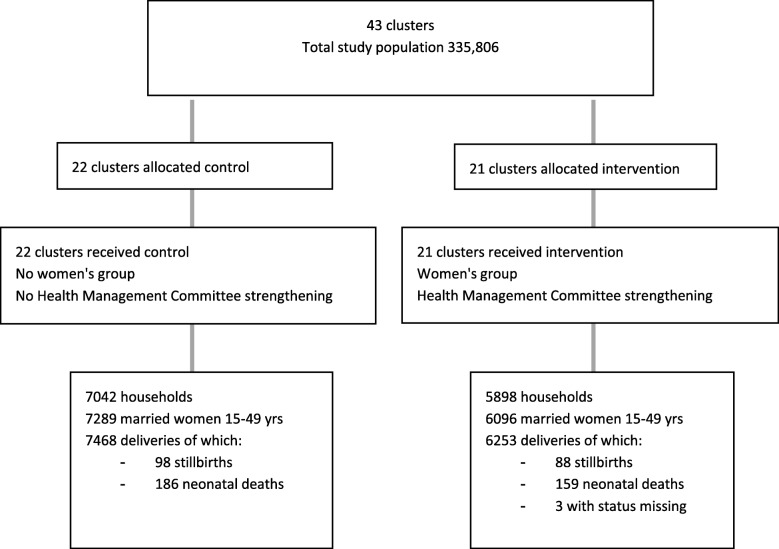
Consort diagram

We conducted an intention to treat analysis including mothers with complete data enrolled from October 1st 2010 to September 30th 2012.. We identified 7468 deliveries in control clusters and 6253 in intervention clusters from 1st October 2010 to 30th September 2012. Figure 5 shows the trial profile. There were 94 stillbirths and 176 neonatal deaths in control and 87 stillbirths and 149 neonatal deaths in intervention clusters (status missing in 3 cases).

### Outcomes and estimation

In the study clusters, only 39% (5403) of deliveries were in health institutions and most were attended by trained health workers. The newborn mortality rate was 23.8 per 1000 in the intervention arm and 23.6 per 1000 in the control arm. The stillbirth rate was 13.9 per 1000 in the intervention arm and 12.6 per 1000 in the control arm. Trained health workers attended only 64 (0.8%) home deliveries (Table 6). There were no differences between control and intervention arms in the primary outcomes of institutional delivery (OR 1.45, 95% CI 0.76 to 2.78) or attendance by trained health workers. There was also no difference in the secondary outcomes of antenatal or postnatal care uptake, neonatal deaths or stillbirths. In intervention areas, institutional deliveries were slightly more likely to be free at the point of care, and women were slightly more likely to receive the maternity incentive. More deliveries were conducted at Sub Health Posts by Auxiliary Nurse Midwives (ANM) in intervention than in control clusters.

**Table 6:** Comparison of allocation groups in the trial period, October 2010 – September 2012

	Control n (%)	Intervention n (%)	Odds ratio (95% CI)	All n (%)
**Primary outcomes**	**7468 (100)**	**6253 (100)**		**13,721 (100)**
Institutional delivery	2875 (38.5)	2532 (40.5)	1.45 (0.76, 2.78)	5407 (39.4)
Home delivery	4590 (61.5)	3721 (59.5)	Ref	8311 (60.6)
Any delivery conducted by doctor, nurse or ANM	2872 (38.5)	2498 (40.0)	1.43 (0.74, 2.74)	5370 (39.1)
Any delivery conducted without a doctor, nurse or ANM	4596 (61.5)	3755 (60.1)	Ref	8351 (60.9)
Institutional delivery conducted by doctor, nurse or ANM	2826 (37.8)	2480 (39.7)	1.43 (0.75, 2.75)	5306 (38.7)
Home delivery conducted by doctor, nurse or ANM	46 (0.6)	18 (0.3)	0.49 (0.20, 1.20)	64 (0.5)
Stillbirth rate (per 1000 births)	98 (13.0)**	88 (14.0)**	1.06 (0.75, 1.50)	186 (13.5)**
NMR (per 1000 live births)	186 (25.1)¶	159 (25.6)¶	1.05 (0.75, 1.47)	345 (25.3)¶
Missing data on any primary outcome	3	0		3
**Secondary outcomes**	**7468 (100)**	**6253 (100)**		**13,721 (100)**
> =4 antenatal care visits	5267 (70.5)	4408 (70.5)	1.04 (0.80, 1.35)	9675 (70.5)
Postnatal care visit	2982 (39.9)	2999 (48.0)	1.77 (0.97, 3.23)	5981 (43.6)
Missing data on postnatal care visits	4	1		5
Delivery site*	**2875 (100)**	**2532 (100)**		**5407 (100)**
Sub-Health Post	238 (8.3)	578 (22.8)		816 (15.0)
Health Post	241 (8.4)	115 (4.5)		356 (6.6)
Primary Health Centre	486 (16.9)	408 (16.1)		894 (16.5)
Government hospital	1590 (55.3)	1279 (50.5)		2869 (53.1)
Private hospital	213 (7.4)	115 (4.5)		328 (6.1)
Institution in other district	66 (2.3)	22 (0.9)		88 (1.6)
Private clinic	41 (1.4)	15 (0.6)		56 (1.0)
Free at point of care	2639 (91.8)	2388 (94.3)		5027 (93.0)
Received maternity incentive	2223 (77.3)	2108 (83.3)		4331 (80.1)
Missing data on costs and incentives	41	16		57
**Delivery attendant**	**7468 (100)**	**6253 (100)**		**13,721 (100)**
Doctor	1165 (15.6)	816 (13.0)		1981 (14.4)
Nurse	1982 (26.6)	1449 (23.2)		3431 (25.0)
Auxiliary Nurse midwife	1886 (25.3)	2090 (33.4)		3976 (29.0)
Health Assistant	12 (0.2)	25 (0.4)		37 (0.3)
Assistant Health Worker	127 (1.7)	196 (3.1)		323 (2.4)
Maternal and Child Health Worker	88 (1.2)	150 (2.4)		238 (1.7)
Village Health Worker	2 (0.0)	10 (0.2)		12 (0.1)
Female Community Health Volunteer	120 (1.6)	159 (2.5)		279 (2.0)
Traditional Birth Attendant	82 (1.1)	23 (0.4)		105 (0.8)
Mother-in-law	1916 (25.7)	1667 (26.7)		3583 (26.1)
Husband	807 (10.8)	1085 (17.4)		1892 (13.8)
Family member	1096 (14.7)	1086 (17.4)		2182 (15.9)
Mother	424 (5.7)	361 (5.8)		785 (5.7)
Neighbour	1999 (26.8)	1671 (26.7)		3670 (26.8)
Natal sister	117 (1.6)	134 (2.1)		251 (1.8)
No attendant at all	265 (3.5)	231 (3.7)		496 (3.6)
Missing data on delivery attendant	3	0		3
**Attended FCHV women’s group**	**353 (4.7)**	**896 (14.3)**		**1249 (9.1)**

* Among women with institutional delivery only ** Numbers in brackets are per 1000 births ¶ Numbers in brackets are per 1000 live births

*ANM* Auxiliary Nurse Midwife; *CI* confidence interval; *NMR* Neonatal mortality rate; Odds ratios are univariable

## Discussion

Our cluster randomised controlled trial of HMC strengthening and community mobilisation through women’s groups sought to increase linkages between health facilities and communities, and enable HMCs to improve management and quality in their health institutions. But our intervention did not increase institutional deliveries or trained health worker attendance at home deliveries. We use process evaluation data and the literature to examine the reasons for this.

### Study limitations

We were unable to maintain the status of our control clusters because of their systematic selection by the Makwanpur District Development Committee (DDC) to receive similar interventions provided by a CBO. From a development perspective, the informed planning of the DDC is encouraging, but it affected our potential to demonstrate impact. Our previous mobilisation of communities through women’s groups also affected some control areas. 78% of women’s groups in the control clusters were active at baseline, indicating that they may not have been true controls. The absence of a true counterfactual makes our study findings difficult to interpret.

Another limitation was that we were not able to implement the HMC strengthening intervention as planned. Most HMCs were unable to hold regular meetings and we found it difficult to support them in a systematic way. It has been noted that the VDC secretary has many duties and low priority is often given to HMC issues [12], and regular HMC meetings and follow-up support are important for the success of locally managed interventions [12, 29, 30].

If most of the clusters in the district were receiving interventions to increase institutional delivery, we might expect the proportion of institutional deliveries and deliveries with a trained health worker to increase across the whole district, but this was not the case. Nationally, there was an annual increase in institutional deliveries from 37% in 2010/11 to 44% in 2011/12. Progress had slowed to 45% in 2012/3 [31].

### National and district policy context

Our interventions were implemented during a period in which there was high national political interest in achieving the MDG targets of reducing infant and maternal mortality through increased skilled attendance and institutional deliveries. The numbers of trained health workers and ANMs increased over the study period in both intervention and control clusters, indicating an effect of national policies. Although this supportive political context may have facilitated an increase in institutional deliveries, trained health workers were actively discouraged from attending deliveries at home and received incentives only for institutional deliveries. In this context it is unlikely that increases in trained health worker attendance at home deliveries could have occurred.

Our study and others indicate the need for a supportive context and leadership to support change [32, 33]. Interventions were well received in clusters in which there were motivated individuals and communities, but constrained by contextual barriers such as a lack of decision-making, disciplinary and budgetary powers at the local level [34, 35]. It was difficult to hold HMC chairpersons to account because they were unelected civil servants. Recent elections and decentralisation of health system governance to elected officials in municipalities presents an opportunity for improved leadership and public accountability, and is likely to be a more favourable environment in which to implement an HMC strengthening intervention [36]. In the federal context of Nepal, systematising regular HMC meetings would enable consistent follow-up on plans and opportunities for community participation. If this was not possible, it would be beneficial to have more review meetings to break-down prioritised goals into smaller achievable actions and enable more frequent reflection on progress. Clarity and formalization of governance roles and responsibilities might help to enforce public accountability systems [37], and is recommended in the decentralised governance context.

Few women’s groups initiated contact with HMCs. This might have been because there was no forum to approach, with members being too busy for meetings, or it is possible that HMC members were not effective in representing the concerns of the population. Where HMCs actively engaged communities, they were more successful in achieving dreams. When HMCs represent elites, public engagement needs to be actively sought to increase their effectiveness [38]. Although there are guidelines for representation of marginalised groups in HMCs [11], such members often found it hard to be effective without ongoing training and support, and without political or social standing and connections [39]. Other studies have found that limited resource mobilisation capabilities have constrained the actions of HMCs [40], and the lack of financial incentives to HMC members may be de-motivating and inhibiting for marginalised members [41]. Without interaction between HMC members and community members, health facilities cannot be publicly accountable.

### FCHV capacity development

FCHVs are not used to facilitating discussions: they are used to providing information and giving health advice and some services. Supervision is important to motivate community health volunteers [42] and maintain the participatory nature of the intervention [43]. Our previous women’s group interventions had a higher supervisor-to-facilitator ratio, more experienced facilitators, and more repetition of meetings to develop skills and confidence. In this intervention FCHVs only had one chance to conduct one meeting per month. Despite these difficulties, supervisors were positive about the intervention and groups enjoyed identifying problems and taking action together. Supervisors felt that FCHVs who had gained most from the intervention would continue their activities, and many felt that the women’s group intervention would be sustained by FCHVs after the trial period.

### Attendance of pregnant women

Although attendance of pregnant women in FCHV-led women’s groups was higher in intervention than control clusters, the proportion of pregnant women attending was relatively low (14% in the intervention arm). Previous meta-analysis has shown that women’s group interventions with high coverage (over 30% of pregnant women attending) have observable effects on neonatal and maternal mortality, whereas interventions with lower coverage show no effects [20]. Although many groups in our study conducted home visits to pregnant women, this may not have had the same effect as attending a group. Our trial adds to the evidence base about the need for pregnant women to participate in groups to increase their effectiveness.

## Conclusion

Our study contributes to health systems research on public accountability mechanisms to improve maternal and newborn survival. Through a cluster randomised controlled trial and concurrent process evaluation we have explored the reasons that our intervention did not show significant increases in trained health worker attendance at delivery or institutional deliveries in intervention clusters when compared with control clusters. Home deliveries accounted for over half of all deliveries in both intervention and control clusters. The national focus and financial support to increase institutional deliveries through recruitment of ANMs and incentives affected the whole district. Additionally, DDC encouragement for other CBOs to focus on control clusters and the residual effect of our previous women’s group interventions prevented our study from having true control areas. We support calls for further research on the impact of health systems interventions [4, 44].

We feel that it is possible to mobilise communities through community health volunteers but recommend that they should be adequately supervised to support skills development and maintain their enthusiasm. By strengthening HMCs, we experienced some success in supporting communities to reduce institutional barriers to maternal care seeking, but in many clusters HMCs did not perform as we expected. Regular follow-up with HMCs might enable more focused planning and reflection on progress, enabled by systematisation of regular meetings. The newly decentralised context of Nepal presents an opportunity to formalise and increase awareness of HMC roles and responsibilities under local governance structures which could enable increased public accountability and enforcement of these responsibilities. More research is necessary to demonstrate how communities and health systems can work together to overcome the multiple barriers faced in accessing institutional deliveries.

## Data Availability

The datasets used and/or analysed during the current study are available from the corresponding author on reasonable request.

## Abbreviations

ANM: Auxiliary Nurse Midwife
CBO: Community Based Organisation
DDC: District Development Committee
FCHV: Female Community Health Volunteer
HMCs: Health Management Committees
MIRA: Mother and Infant Research Activities
VDC: Village Development Committee
